# Pharmacotherapy of chronic obstructive pulmonary disease: Therapeutic considerations with a focus on inhaled corticosteroids

**DOI:** 10.4102/safp.v62i1.5198

**Published:** 2020-12-08

**Authors:** Elvis M. Irusen, Thandeka D. Malange

**Affiliations:** 1 Division of Pulmonology, Department of Medicine, Faculty of Medicine and Health Sciences, Stellenbosch University, Cape Town, South Africa; 2 Tygerberg Hospital, Cape Town, South Africa

**Keywords:** bronchodilators, chronic obstructive pulmonary disease, guidelines, inhaled corticosteroids, pharmacotherapy

## Abstract

International and national guidelines on chronic obstructive pulmonary disease (COPD) emphasise bronchodilators as first-line therapy. However, in considering them the ‘foundation’ of treatment, attention has shifted from the fact that COPD is fundamentally an inflammatory disease. The mainstay ought to be anti-inflammatory medication, and inhaled corticosteroids (ICS) are the best agents we have presently. There was initial scepticism about their role, but ICS were subsequently shown to have numerous anti-inflammatory effects. They are synergistic with bronchodilators at a molecular and clinical level and unequivocally improve dyspnoea, quality of life, exacerbation frequency and, more recently, mortality. These benefits are most apparent in the COPD eosinophilic phenotype. These beneficial effects have been met with some reservations because of the predisposition to pneumonia of ICS. This must be seen in context: over 90% of COPD patients in all clinical trials do not get pneumonia. The fact that patients with COPD are predisposed to pneumonia because of the disease itself is disregarded; this is a crucial omission as this constitutes the baseline incidence of about 3%. When one allows for this, then in the clinical reports, the excess risk of pneumonia ranges from zero to a maximum of 3%. Equally, some of the systemic effects attributed to ICS fail to appreciate that the disease, smoking and older age are risk factors in themselves, and ICS do not aggravate these. Chronic obstructive pulmonary disease has considerable impact on respiratory reserve and is associated with increasing morbidity; optimal outcomes are best achieved with long-acting bronchodilators and ICS co-prescription.

## Introduction

Chronic obstructive pulmonary disease (COPD) has classically encompassed the pathological entities of emphysema and chronic bronchitis. However, in the last decade these terms are no longer included in the formal definition; instead, national and international guidelines have defined it spirometrically,^1,2^ to the extent that the pathology receives scant attention. Doctors have progressively been advised to manage patients exclusively through spirometry, symptoms and exacerbations with no consideration of the underlying disease process.^3^ Indeed, in the most recent guideline, the pathology is not mentioned at all.^3^

The best practice guidelines recommend that:^1,2,3^

- The cornerstone of treatment is bronchodilators (BD) with the possibility that inhaled corticosteroids (ICS) will never be included in the life course.
- Treatment with ICS should be avoided as:
  ■ The inflammation present in the pathology of COPD is ICS-resistant.
  ■ They are associated with the development of pneumonia.
- Only when the disease is severe and/or an associated eosinophil phenotype, then ICS may be utilised.
- If the patient is stable on an ICS–bronchodilator regimen, then the ICS must be withdrawn.^1,3^

Herein, the above claims are dissected via reviewing the data on BD alone as the foundation of treatment and the apparently minor role of ICS. The validity of evidence regarding pneumonia and the issue of steroid withdrawal will also be discussed.

All the data that will be reviewed are derived only from smoking-related COPD. Other important aspects such as smoking cessation and rehabilitation will not be expounded.

## Limitations of spirometry in chronic obstructive pulmonary disease

It must be the supreme paradox. Chronic obstructive pulmonary disease is defined as a spirometrically irreversible disease, and subjects are then given BD to see how much the lung function can improve. Chronic obstructive pulmonary disease as a disease is not being understood. It is not asthma, where smooth muscle hyperplasia and excessive bronchoconstriction occur and which is very responsive to BD. This is not the bronchial pathology of COPD, which manifests as irreversible structural changes with persistent airway narrowing, inflammation and mucus gland hypersecretion. One must therefore appreciate that monitoring lung function serially to assess response to treatment is actually severely limited. This is one of the reasons why the effect of ICS cannot be appreciated – it cannot be monitored appreciably with spirometry. With remodelling and a somewhat fixed airflow limitation, even the most potent anti-inflammatory agent will not result in substantial changes in lung function. Despite these constraints, up to 60% of COPD subjects may show spirometric reversibility as defined by the American Thoracic Society.^4,5^

The Global Initiative for Obstructive Lung Disease (GOLD) and South African guidelines consider the definition for COPD exemplified by the post-bronchodilator forced expiratory volume in one second (FEV_1_)/forced vital capacity (FVC) of < 0.70 as being diagnostic.^1,2^ Another limitation, however, is that the criterion is neither sensitive nor definitive for the detection of COPD.^4^ The SPIROMICS (Subpopulations and Intermediate Outcome Measures in COPD Study) research group reported on an observational study involving 2736 current or former smokers.^6^ Respiratory symptoms were present in 50% of subjects with spirometry in the normal range (who would not have met the COPD definition). These participants had:

- a mean rate of exacerbations per year that was significantly higher than that of asymptomatic smokers (0.27 vs. 0.08, *p* < 0.001)
- a greater limitation of activity, mildly reduced FEV_1_, FVC and inspiratory capacity and airway wall thickening on high resolution computerised tomography (HRCT) than the asymptomatic group
- been taking BD (42%) and ICS (23%).

These data suggest that we need to be circumspect about the GOLD definition, carefully evaluate at-risk patients for symptoms and treat them appropriately.

A disconnect also exists between spirometry and the severity of COPD. Even in mild disease, as defined by FEV_1_ of 80% or greater of the predicted value, most transitional bronchioles and about a third of the alveolar surface of the lung have already been destroyed.^7^

## Inflammation, corticosteroids and clinical effects in chronic obstructive pulmonary disease

Inflammation is present in the early stages of disease, is known to be neutrophil-driven and leads to chronic bronchitis and damage to the lung parenchyma. Inhaled corticosteroids are known not to have a major antineutrophil effect.^8^ Early studies on the anti-inflammatory actions of ICS in COPD demonstrated that they were not as rapid as in asthmatics, dissuading clinicians. However, when these were studied over extended periods, multiple anti-inflammatory effects were noted.^9^ One reason for the limited responsiveness to ICS has also been ascribed to reduced histone deacetylase 2 (HDAC2) expression.^10^ It was subsequently found that certain anti-inflammatory effects of steroids are independent of HDAC2.^11^ Thus, this pathway can be bypassed for ICS to ameliorate tissue damage. A meta-analysis of studies that evaluated inflammatory biomarkers in sputum, bronchoalveolar lavage fluid and biopsy specimens demonstrated that ICS led to a reduction in cluster of differentiation 4 (CD4+), CD8+, neutrophils and lymphocytes.^12^ Another study concluded that even with persistent smoking, long-term ICS treatment could still produce anti-inflammatory effects in the lung.^13^

A Dutch group has done extensive studies of ICS in COPD.^14,15^ In one, with fluticasone propionate (FP) and salmeterol, almost 300 genes involved in the pathogenesis exhibited altered expression after treatment.^15^ Clinically, the FEV_1_ in every patient improved pari passu with the ICS, but this ranged from poor to good. As in all studies, this Gaussian gradation of responses reflects the variability of steroid responsiveness of the pathogenetic mechanisms and the capacity for bronchodilator reversibility from the remodelling process.

The synergy between molecular interactions of ICS and long-acting beta agonists (LABA) are well recognised in asthma and occur in COPD as well; certain *in vitro* effects of high-dose ICS could be achieved with lower doses of ICS when combined with LABA.^16^

Clinically, the addition of ICS to BD results in a relative improvement in lung function, quality of life and exacerbation reduction – all to the order of approximately 25%.^17^ Exacerbations are particularly important. These can be life threatening, are major cost-drivers and can result in acute permanent deterioration in the trajectory of functional decline. Hence, this is a major end point in clinical studies, and it is only supplementary ICS that influence this substantially. Because exacerbations tend to occur in those with prior such episodes and more severe disease (FEV_1_ < 60%), this group has been the focus of study. However, even those without such an experience and FEV_1_ of > 60% may also benefit from ICS. This was observed in the Study to Understand Mortality and Morbidity in COPD (SUMMIT) sub-study and the Efficacy and Safety of Triple Therapy in Obstructive Lung Disease (ETHOS) clinical trial.^18,19^

The only other agent to address inflammation that has shown potential is the phosphodiesterase-4 inhibitor roflumilast. It has demonstrated a reduction in COPD exacerbations with improvement in the quality of life (QOL) and lung function.^1^ It is the final choice in the GOLD therapeutic algorithm, indicated only in chronic bronchitis, but some side effects may limit its use.

## Adverse effects

### Metabolic and ocular effects

The universal use of ICS has been tempered by the possibility of adverse effects and pneumonia. Alterations in bone mineral density, fractures and the development of cataracts have been reported. However, at least two major studies, Towards a Revolution in COPD Health (TORCH) and ETHOS, failed to show any increased risk between the steroid arms and the bronchodilator and placebo arms (the latter reflecting the innate risk) from ICS.^17,19^ A Cochrane review also found no deleterious effect on bone mineral density in COPD subjects.^20^ Smoking, advancing age and oral corticosteroids for exacerbations are confounders when considering these complications.

### The risk of pneumonia

Pneumonia, usually non-fatal,^17,19^ is recognised as an adverse effect of ICS therapy in COPD, and recent research has been careful to emphasise and monitor for the development of this complication. However, it is this introspection that has revealed a curiosity. In several studies with large numbers of patients who were potentially at risk, the incidence of pneumonia was not significantly different to the placebo or bronchodilator groups.^21,22,23,24,25^ There is no evidence that BD cause pneumonia; hence these latter two groups represent the baseline risk of pneumonia in COPD.

The primary SUMMIT study consisted of > 16 000 subjects with just over 4000 participants in each of four groups (fluticasone furoate–vilanterol [FF/VI], FF, VI and placebo).^21^ There was no statistically significant difference in pneumonia events between the steroid and the LABA or placebo arms. This begs the question of what the possible reasons for pneumonia are. Pneumonia is a feature of airway dysbiosis, diminished bronchopulmonary immune protection and systemic depressed immunity. Smoking alters the airway microbiome and depresses cilial and bronchial immunity.^26^ More severe COPD is encountered in older individuals with a waning immunity, and those with exacerbations are prescribed oral or parenteral corticosteroids, which also have an immunosuppressant effect. These factors are responsible for the baseline risk of pneumonia,^27^ which the research suggests is approximately 2% – 3%.^21,22,23,24,25^ Thus if one interrogates the pharmaceutical studies and deducts this baseline risk (placebo and bronchodilator groups) from those given ICS, the excess risk is in the range of 0% – 3%, that is, no or very low predisposition, and it is apparent that the ICS confers no or minimal additional predilection (Table 1).^21,22,23,24,25,27^ One of the primary reasons to prescribe ICS is to reduce the risk of exacerbations. The European Medicines Agency’s Pharmacovigilance Risk Assessment Committee (PRAC) has recommended that ICS can continue to be used as the benefit–risk profile is favourable (greater exacerbation reduction vs. few pneumonia events).^28^ There has also been controversy as to whether different ICS have a different propensity to pneumonia; the agency stated that this is a class effect and could not find differences between the various glucocorticoids.

**TABLE 1:** Studies in which inhaled corticosteroids conferred no additional risk of pneumonia compared to placebo or bronchodilator.

Study	Corticosteroid
SUMMIT, 2016	Fluticasone furoate
WISDOM, 2014	Fluticasone propionate
TRIBUTE, 2018	Beclomethasone dipropionate
KRONOS, 2018	Budesonide
SUNSET, 2018	Fluticasone propionate

## The eosinophilic phenotype in chronic obstructive pulmonary disease

For the COPD participants, having higher eosinophil counts in blood or sputum – an ‘eosinophilic phenotype’ – was noted to confer ICS responsiveness.^29,30^ It is now generally accepted that COPD patients with eosinophilia (≥ 2% eosinophils or ≥ 150 eosinophils/*µ*L) respond better to ICS therapy. A post hoc analysis of the Investigating New Standards for Prophylaxis in Reduction of Exacerbations study (INSPIRE) – which utilised a 2% eosinophil cut-off – demonstrated that FP/salmeterol was linked with a 25% exacerbation risk reduction compared to tiotropium alone.^31^

In the WISDOM study, dual long-acting BD and maintenance FP exhibited a linear relationship of further exacerbation reduction with higher eosinophil counts compared to dual BD alone – in other words, the higher the eosinophil count, the better the exacerbation reduction.^32^ In another, the Study to Understand the Safety and Efficacy of ICS Withdrawal from Triple Therapy in COPD (SUNSET), patients with high eosinophils went on to experience the largest lung function loss and exacerbation risk, prompting the authors to state that in this group, ICS should not be discontinued.^23^

## Combination treatment and advent of single inhaler triple therapy

The progressive nature of COPD, intractable symptoms and the increasing propensity to exacerbations mean that there is a need to sequentially add treatment. The early options were a long-acting beta agonist (LABA), and/or a long-acting muscarinic antagonist (LAMA), as these are superior to their short-acting counterparts, and ICS in various combinations and devices. An ICS/LABA is superior to LAMA monotherapy.^33^ The need for all three agents and short-acting agents for rescue could mean up to four different inhalers. Such polypharmacy does not aid adherence. Moreover, COPD and age are accompanied by increasing frailty, difficulty in inhalation techniques and coordination. As a proof of concept, multiple inhaler triple therapy was investigated. In the Lung Function and Quality of Life Assessment in COPD with Closed Triple Therapy (FULFIL) trial, umeclidinium (Umec) was added to FF/VI and resulted in a significant approximately 180-millilitres (mL) FEV_1_ net gain and QOL improvement when compared to budesonide (Bud)/FF at the one year mark.^34^ Single inhaler triple therapy (SITT) was then embarked upon, with all consistently showing improvements in lung function, QOL measures and exacerbation reduction. In TRIBUTE, a 15% reduction in exacerbation rate was noted with extra-fine beclomethasone dipropionate, formoterol furoate and glycopyrronium (G) bromide (BDP/Form/GB) compared with indacaterol/GB.^28^ Higher rate reductions were demonstrated in the other SITT studies, IMPACT (FF/Vi/Umec) and ETHOS (Bud/Form/G) (Table 2).^23,35^

**TABLE 2:** Illustration of the effect of inhaled corticosteroids added to bronchodilators on reduction of exacerbations in chronic obstructive pulmonary disease.

Study	ICS/LABA/LAMA	ICS/LABA	LABA/LAMA
**ETHOS**	Budesonide Formoterol/glycopyrrolate	Budesonide Formoterol	Formoterol Glycopyrrolate
Annual exacerbation rate Rate reduction	1.07 24% lower: rate ratio, 0.76; 95% CI, 0.69–0.83; *p* < 0.001	1.24 13% lower: rate ratio, 0.87; 95% CI, 0.79–0.95; *p* = 0.003	1.42
**IMPACT**	Fluticasone furoate Vilanterol/umeclidinium	Fluticasone furoate–vilanterol	Vilanterol Umeclidinium
Annual exacerbation rate Rate reduction	0.91 0.75; 95% CI, 0.70–0.81; 25% difference; *p* < 0.001	1.07 0.85; 95% CI, 0.80–0.90; 15% difference; *p* < 0.001	1.21
**TRIBUTE**	Beclomethasone Formoterol/glycopyrronium	Not studied	Indacaterol Glycopyrronium
Annual exacerbation rate Rate reduction	0.5; 95% CI, 0.45–0.57 0.848; 95% CI, 0.723–0.995, *p* = 0.043		0.59; 95% CI, 0.53–0.67

Note: The different studies should not be compared as there were different designs and patient populations. Comparison with LABA/LAMA are shown.

ICS, inhaled corticosteroids; LABA, long-acting beta agonists; LAMA, long-acting muscarinic antagonist; 95% CI, 95% confidence interval.

## Mortality benefit

A mortality benefit is the most sought-after goal in COPD. It was hoped that an agent that was potentially ‘disease modifying’, such as ICS, would prove beneficial over BD. An early study showed a numerical benefit, but this did not reach statistical significance (TORCH).^21^ This threshold was finally passed with the IMPACT study, when the ICS (FF), either with dual BD (umeclidinium and vilanterol) or vilanterol (FF/VI), proved superior to the dual BD.^35^ This is likely a result of FF – one of the most recent glucocorticoids to be developed – which has the highest receptor-binding properties of all ICS with pronounced anti-inflammatory effects and a reduction in exacerbations. The only other trial to show a mortality benefit was ETHOS, utilising high-dose budesonide and glycopyrronium–formoterol.^23^

## Withdrawal of the corticosteroid component in a dual or triple regimen containing inhaled corticosteroids

There is an emerging strategy that when the disease is stable, then the ICS component in either a dual or triple treatment regimen, because of the perceived risks, can be discontinued^36^; this is strongly discouraged. It also stems from a parallel in asthma where a reduced ICS dose is required when the inflammation subsides. This is not true of COPD, as the ICS is maintaining stability of the disease. In the development of COPD medication, dual therapy including ICS was first introduced and became the standard of care. With the caution around ICS, studies were then performed where the ICS was withdrawn. All consistently showed that that this was deleterious; patients rapidly experienced exacerbations,^37,38^ a persistent decrease in lung function^39^ and lowered quality of life.^37^ As anticholinergics became available, these were progressively added to the ICS + LABA: triple therapy. An attempt was again made to omit the ICS. A salutary landmark study showed that this was an unwise decision. In the WISDOM study, subjects on tiotropium, salmeterol and fluticasone propionate (FP, 1000 microgram [*µ*g]/d) gradually had the FP withdrawn. In those maintained on dual BD only, the FEV_1_ immediately and progressive declined over one year, as did the QOL, and was not equivalent in exacerbation protection. In the SUNSET study, lung function deteriorated in those maintained on indacaterol–glycopyrronium only compared to those on tiotropium and salmeterol–FP.

A second major reason not to discontinue ICS is a failure to appreciate the natural history of COPD. The clinical course of COPD is characterised by accelerated decline in lung function and concomitant functional capacity, as was elegantly demonstrated by the famous graph of Fletcher and Peto.^40^ Because of this trajectory, therapy needs to be augmented progressively in COPD rather than withdrawn. In general, more treatment is required to improve symptoms and prevent exacerbations with the passage of time. In a general practice report, 30% of newly diagnosed patients in primary care had to progress to triple therapy (dual BD + ICS) within two years of presentation.^41^

## Conclusion

The augmentation of clinical improvements and course of the disease with ICS when added to mono or dual BD clearly attest to the importance of suppressing inflammation and indicate that this component should not be forgotten. This effect is particularly pronounced in exacerbation reduction. The hesitancy in prescribing ICS because of the possibility of adverse metabolic effects and pneumonia, although important, is unwarranted. When confounders for these occurrences have been considered, the risks appear negligible or of very low frequency at worst. The data also show that ICS withdrawal is deleterious and ill-advised in a disease with a natural history of progressive decline necessitating increased pharmaceutical support. Those patients with more eosinophils should be targeted for early ICS therapy, and de-escalation should not be contemplated for them. The pharmacotherapy discussed is available in several formulations singly and in combination, with varying extent of availability and cost in different countries. The clinician should be prudent in choice, preferably choosing an ICS in the regimen. When the disease has progressed beyond the mild stage, dual BD and ICS, ideally in a single inhaler, are a compelling option for optimal therapeutic outcomes.
